# First Report on Genome Editing via Ribonucleoprotein (RNP) in *Castanea sativa* Mill.

**DOI:** 10.3390/ijms23105762

**Published:** 2022-05-20

**Authors:** Vera Pavese, Andrea Moglia, Silvia Abbà, Anna Maria Milani, Daniela Torello Marinoni, Elena Corredoira, Maria Teresa Martínez, Roberto Botta

**Affiliations:** 1 Dipartimento di Scienze Agrarie, Forestali e Alimentari-DISAFA, Università degli Studi di Torino, Largo Paolo Braccini 2, Grugliasco, 10095 Torino, Italy; vera.pavese@unito.it (V.P.); silvia.abba@edu.unito.it (S.A.); annamaria.milani@unito.it (A.M.M.); daniela.marinoni@unito.it (D.T.M.); roberto.botta@unito.it (R.B.); 2 Misión Biológica de Galicia, Sede de Santiago, Consejo Superior de Investigaciones Científicas, Avd. Vigo, s/n, 15705 Santiago de Compostela, Spain; elenac@mbg.csic.es (E.C.); temar@mbg.csic.es (M.T.M.)

**Keywords:** CRISPR/Cas9, European chestnut, protoplast, transgene-free, phytoene desaturase

## Abstract

*Castanea sativa* is an important tree nut species worldwide, highly appreciated for its multifunctional role, in particular for timber and nut production. Nowadays, new strategies are needed to achieve plant resilience to diseases, climate change, higher yields, and nutritional quality. Among the new plant breeding techniques (NPBTs), the CRISPR/Cas9 system represents a powerful tool to improve plant breeding in a short time and inexpensive way. In addition, the CRISPR/Cas9 construct can be delivered into the cells in the form of ribonucleoproteins (RNPs), avoiding the integration of exogenous DNA (GMO-free) through protoplast technology that represents an interesting material for gene editing thanks to the highly permeable membrane to DNA. In the present study, we developed the first protoplast isolation protocol starting from European chestnut somatic embryos. The enzyme solution optimized for cell wall digestion contained 1% cellulase Onozuka R-10 and 0.5% macerozyme R-10. After incubation for 4 h at 25 °C in dark conditions, a yield of 4,500,000 protoplasts/mL was obtained (91% viable). The transfection capacity was evaluated using the GFP marker gene, and the percentage of transfected protoplasts was 51%, 72 h after the transfection event. The direct delivery of the purified RNP was then performed targeting the *phytoene desaturase* gene. Results revealed the expected target modification by the CRISPR/Cas9 RNP and the efficient protoplast editing.

## 1. Introduction

The European chestnut (*Castanea sativa* Mill.) is a multipurpose tree that offers a wide range of secondary products and ecosystem services and is recognized worldwide for its excellent wood and nut quality [1,2]. In spite of the growing demand for nuts by the confectionery industry and the fresh market, there are constraints that hinder the renewal and the new planting of orchards in many areas of Europe.

Nowadays, there is a growing interest in developing breeding programs to provide improved cultivars that increase yield and nut quality, with better adaptability to climate change and tolerance to pathogens and pests. Chestnut is highly susceptible to two severe diseases that threaten its survival: ink disease caused by the oomycete *Phytophthora* spp. and chestnut blight caused by the fungus *Cryphonectria parasitica* [3]. In addition, chestnut is affected by the Asian gall wasp *Dryocosmus kuriphilus* Yasumatsu (Hymenoptera Cynipidae), an invasive insect that causes gall formation, found in Europe, in northwestern Italy, for the first time in 2002 [4,5]. Finally, the nut rot and canker agent *Gnomoniopsis castaneae* G. Tamietti [6] has become a serious problem for nut quality. Genes responsible for plant–pathogen compatibility, such as susceptibility genes [3], can be detected and used in target silencing programs to improve plant tolerance.

The improvement of woody fruit species through traditional breeding techniques has several limitations mainly caused by their high degree of heterozygosity, the length of their juvenile phase, the long generation times and auto-incompatibility systems [7].

Molecular biology, genome sequencing and genetic engineering offer innovative strategies to improve plant knowledge and confer valuable genetic traits to elite genotypes in order to overcome the challenges of the XXI century: to produce more with less, overcome the risk of food reduction due to climate change and increase yield in a sustainable manner [8].

New plant breeding techniques (NPBTs) represent a valid strategy to quickly improve plant breeding [9]. Currently, among NPBTs, the CRISPR/Cas9 (Clustered Regularly Interspaced Short Palindromic Repeats-Cas9) technique is considered one of the most effective low-cost tools for plant genetic engineering [10].

In chestnut, efficient protocols of genetic transformation were set up [11,12,13], and the first example of CRISPR/Cas9 technology in the *Castanea* genus was recently published by Pavese et al., 2021 [14].

Even if there is a growing interest in applying the CRISPR/Cas9 system to woody plants in order to rapidly generate ideal cultivars deprived of negative or undesired genetic traits [15], this technique is still limited due to recalcitrance to de novo organogenesis and the low transformation efficiency of these species.

To date, the CRISPR/Cas9 technology has been applied to a small number of woody species such as sweet orange [16], poplar [17], strawberry [18], apple [19], pear [20], grapevine [9], cacao [21] and chestnut [14].

The CRISPR/Cas9 complex is usually delivered using *Agrobacterium tumefaciens* or through particle bombardment; the complex thus can be integrated into the plant genome with the drawback that both the Cas9 enzyme and the gRNA can remain active for a long time, causing off-target events.

To avoid transgene integration, the CRISPR/Cas9 construct can be delivered as a ribonucleoprotein (RNP) form, which has the advantage of minimizing or even avoiding the stable integration of recombinant DNA. In this case, the components of the CRISPR/Cas9 system are in vitro synthesized, pre-assembled and then delivered into the plant cell protoplasts as RNP [22]. The RNP-based system is efficient because it immediately acts on the target site without requiring the activation of the transcription process, and then RNPs are rapidly degraded [23] thanks to the natural cellular mechanisms of protein and RNA turnover [22]. Moreover, the protoplast is a useful matrix for genetic transformation due to its permeability to exogenous DNA molecules, and the de novo organogenesis from a single cell allows for maintaining genetic uniformity [15]. While the transfection efficiency of protoplasts is quite high [24], the polyethylene glycol (PEG)mediated delivery method is limited in the ability to isolate high-quality and viable protoplasts and the subsequent establishment of suspension cells. Plant regeneration from protoplasts remains unestablished in many plant species, being especially difficult in woody species; in addition, somaclonal variation and genome instability were reported in regenerated lines [25].

CRISPR RNP-based genome editing offers the opportunity to produce edited plants by means of DNA-free approaches, opening new perspectives for breeding purposes and potentially better acceptance by consumers as compared to classic GMOs (genetically modified organisms) [26]. In spite of these advantages, this technology has scarcely been applied so far to woody species: from the literature, we can only mention the reports on apple [15,27], grapevine [27] and pine [28].

Since there is no evidence of gene editing using RNPs in *Castanea sativa*, here we present: (i) the first protoplast isolation and transformation protocol, based on the use of the green fluorescent protein (GFP) as a marker gene; (ii) the first example of the direct delivery of CRISPR/Cas RNP to chestnut protoplasts by targeting the *phytoene desaturase* (*pds*) gene, which is involved in chlorophyll biosynthesis [14].

## 2. Results and Discussion

### 2.1. Protoplast Isolation from Somatic Embryos

Embryogenic calli are an excellent starting material for protoplast isolation, as previously underlined in grape and apple [27,29]. Being a very friable matrix, they are easily disaggregated into small pieces, unlike the chestnut leaf tissue, which has a higher cellulose and lignin content and is rich in phenols that could be deleterious for enzyme activities [30,31]. Moreover, an embryogenic callus can produce a higher percentage of regenerable protoplasts as compared to somatic tissues [29].

During protoplast isolation, several parameters affect yield and extraction quality, including the selection of the starting plant material, the enzyme concentrations and the incubation time in the enzymatic mixture [32]. Protoplasts were isolated from *Castanea sativa* embryogenic calli using an enzymatic digestion mixture that involved the use of cellulase R-10 (1%) and macerozyme R-10 (0.5%). The final protoplast yield, evaluated by counting the cells with a hemocytometer, was 4,500,000 protoplasts/mL. The number of isolated protoplasts was comparable to those obtained in experiments performed in grape and apple [27,29], and higher than the numbers reported in *Quercus ilex* and *Populus alba* [30]. During protoplast isolation, it is pivotal to obtain healthy, viable cells without a large number of nonviable cells; the addition of the sucrose gradient allows the removal of the broken nonviable protoplasts from the final mixture.

**Figure 1:**
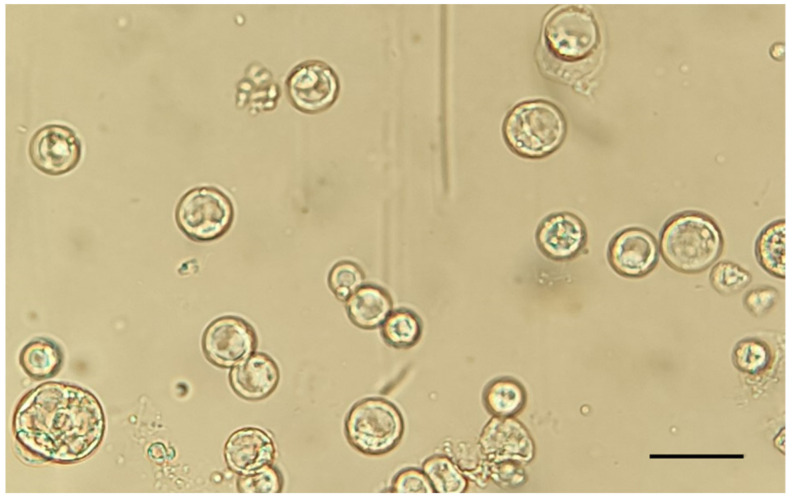
Extracted protoplasts. Magnification 40×. Scale bar = 100 µm.

The protoplast viability was tested using trypan blue staining, which colors non-viable protoplasts in blue. The trypan blue assay showed a high viability percentage (91 ± 1%), in accordance with previous studies [27,33]. The enzymatic solution recipe and the incubation times adopted for chestnut embryogenic calli were demonstrated to be effective; the obtained protoplasts showed a perfect spherical shape and a 20–70 μm diameter size, and no aggregates of undigested cells were detected (Figure 1).

### 2.2. Protoplast Transfection with GFP Vector

GFP protein is an excellent marker to test the transformation efficiency for the first time in a new plant species. Several species have been transformed using GFP marker gene, including *Elaeis guineensis* [34], *Brassica oleracea* [35] and *Cucumis sativus* [36]. As a first step in the development of an efficient transformation protocol in chestnut, we used GFP as a visual marker for protoplast transfection.

PEG-mediated transfection, thanks to its simplicity and low-cost application, is a standard method to introduce DNA into protoplasts and has been used in several plant species [32,37,38]. The plasmid DNA containing the GFP marker complex penetrates directly into the protoplast cell by direct absorption, thanks to the PEG action, which makes the cell membranes permeable to DNA.

**Figure 2:**
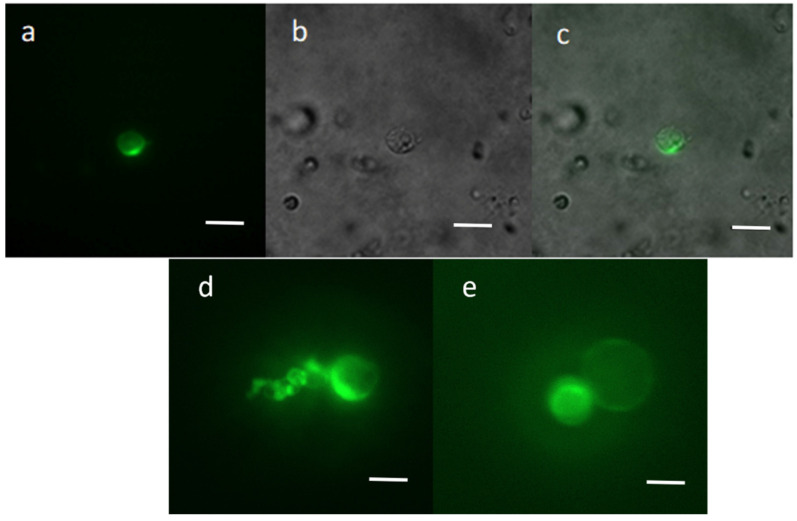
Protoplasts transfected with the pAVA393 plasmid containing the GFP expression cassette. The GFP signal was detected by fluorescence microscopy 72 h after the transfection event, under blue light (**a**,**d**,**e**), white light (**b**) and fusion of the two images (**c**). Scale bar = 100 µm.

Recombinant vector pAVA393:GFP was transferred into chestnut protoplasts, and the GFP transfection efficiency was evaluated 72 h after the transfection event using a Nikon Eclipse Ti2 fluorescence microscope. The results revealed good protoplast integrity, with cells showing an intact and spherical shape even after 72 h from the transfection event. Fifty-one percent of the protoplasts showed the GFP expression (Figure 2), which was observed in intracellular compartments. The absence of the signal in the negative control (empty vector) confirmed the transfection success.

In order to obtain reproducible results, high transfection values (>50%) are needed. The GFP expression rate depends on the GFP plasmid DNA quality and on the ratio between the plasmid DNA and the viable protoplast numbers [37].

### 2.3. Protoplast Transfection with CRISPR RNPs

Genome editing may represent the future of breeding in woody species that present high genome complexity and a long juvenile phase. Thanks to genome editing techniques, it is possible to perform target mutations in order to increase key agronomic traits in a shorter time. Due to the construct integration within the genome, products developed by gene editing and other new genetic technologies must be subjected to GMO regulations in many countries [26,39]. For this reason, researchers are trying to develop new strategies to circumvent DNA integration, such as with the CRISPR/Cas9 delivery into protoplasts using RNP [28], a complex consisting of the recombinant Cas9 nuclease and the CRISPR RNA (crRNA) transcribed in vitro.

In this paper, we report on the first example of transgene-free transfection in chestnut protoplasts. To determine whether the CRISPR/Cas9 system may be suitable for gene editing in *C. sativa*, we used as a target the *pds* gene. We adopted the same gRNA adopted in our previous work [14] targeting the Amino_oxidase domain of PDS.

Total genomic DNA was extracted from transfected protoplasts (samples P1, P2 and P3). In order to detect *pds* gene editing efficiency and the types of mutations, the Sanger sequencing was used in association with TIDE software. Molecular data demonstrated comparable editing efficiency (from 15 to 21%) in the three analyzed samples (Table 1). No mutations were detected in sgRNA-only transfected protoplasts. The most common mutations in our transfected protoplasts were represented by a single nucleotide insertion followed by deletions of one and three nucleotides. Previous observations showed that small indels are the predominant mutations introduced in plants by gene editing [40].

In previous reports, the mutation efficiency derived from CRISPR/Cas9 technology was highly variable, depending on the transformation method. The editing efficiency underlined in our study (~18%) is comparable to data shown by other studies using the RNP complex in *Arabidopsis* (16%) and rice (8.4–19%), and higher than that observed in grapevine (0.1%) and apple (0.5–6.9%) [22,27]. This result is lower than what was observed in CRISPR/Cas9 mediated transformation of chestnut somatic embryos using the same gRNA (~61%) [14]. The gRNA with a mutation rate higher than 10% in protoplast is considered a suitable candidate for recovery of edited plants using other methods [31].

### 2.4. Regeneration of Protoplasts

The protoplasts themselves are only useful for the analysis of cellular functions, and the regeneration of whole plants is necessary to determine how genes affect plant physiology or development. This is a major bottleneck in many plant species and regeneration of trees from protoplasts has resulted in limited success [41,42].

Protoplasts transfected with pAVA393:GFP were incubated in three different regeneration media (named C1, C2, C3). C1 and C3 media, based on MS medium [43], were not suitable for the development of embryogenic calli from protoplasts. The C1 medium was used by Corredoira et al. [44] to obtain somatic embryo induction from leaf explants; the C3 medium was used by Corredoira et al. [45] for somatic embryo induction from immature seeds.

**Figure 3:**
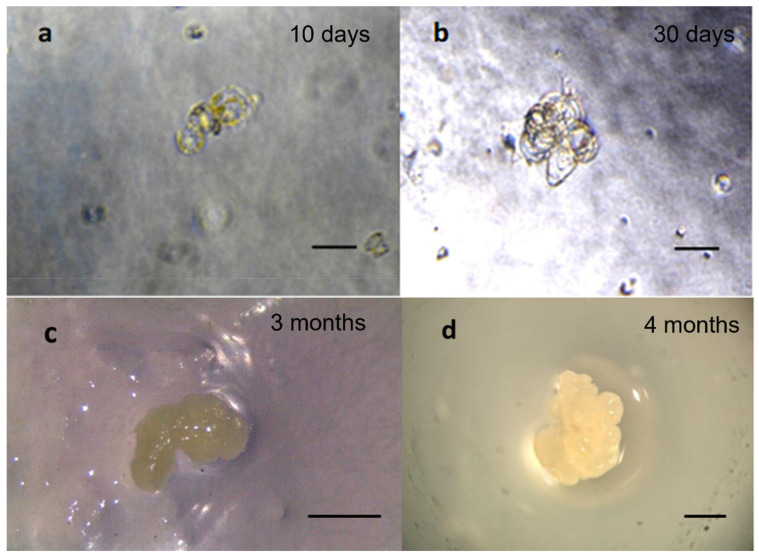
General overview of protoplast development into embryogenic callus in European chestnut observed on C2 medium. (**a**) First cellular divisions after 10 days; (**b**) microcolonies after 30 days; (**c**) embryogenic callus after 3 months of culture; (**d**) embryogenic callus after 4 months. Observations were obtained using the stereomicroscope Leica-Wild Heerbrugg M8. Scale bar = 1 mm.

The best results were observed when protoplasts were cultured on C2 media (Figure 3) containing the Nitsch’s medium [46] supplemented with 1 mg/L naphthaleneacetic acid (NAA) and 0.5 mg/L benzylaminopurine (BAP), and that was used by Bertini et al. [29] for protoplast cultivation of grapevine. The auxins, usually in combination with a cytokinin at low concentration, are the most important components of the culture medium during somatic embryo induction [31]. NAA in combination with BAP has also been used for the induction of somatic embryos in different oak species [47,48,49] belonging to the same *Fagaceae* family as the chestnut. Another factor to consider during somatic embryogenesis induction is the mineral formulation. The reduced macronutrient concentration in the Nitsch’s medium, compared to MS medium, showed a considerable effect on enhancing the organogenesis process. In *Picea glauca*, it was demonstrated that a reduced salt concentration positively influences protoplast development into embryos [41,50].

**Figure 4:**
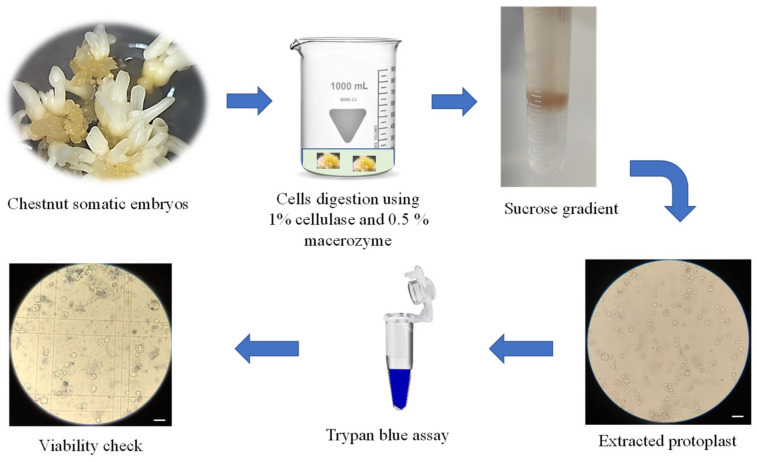
Chestnut protoplast isolation protocol starting from embryogenic callus derived from somatic embryos. Scale bar = 100 µm.

The development of embryogenic tissue from protoplasts in chestnut was slow. The first cell divisions occurred after 10 days (Figure 3a) and microcolony formation was observed after 30 days on the C2 medium (Figure 3b) composed of Nitsch medium. Figure 3 shows the embryogenic callus obtained after 3 (Figure 3c) and 4 (d) months on C2 medium. The embryogenic callus shows a white aspect with a size that in 1 month doubled from 1 mm to about 3 mm.

## 3. Material and Methods

### 3.1. Plant Material

Embryogenic calli of *Castanea sativa* were chosen as starting material for setting up the protoplast isolation protocol. Embryogenic tissue cultures were initiated starting from immature seed cultures as described by Corredoira et al. [45]. Briefly, immature seeds were surface-sterilized and cultured on Murashige and Skoog medium (MS) [43] supplemented with 0.5 mg/L 2,4-dichlorophenoxyacetic acid (2,4 D) and 1 mg/L BAP.

Explants were incubated for 2 months in dark conditions and then transferred to MS medium containing a reduced concentration of BAP (0.1 mg/L) and kept in the growth chamber with a 16/8 h photoperiod, 23 °C temperature. After approximately 3 months, the embryogenic calli were obtained and used as starting materials for setting up the protoplast isolation protocol.

### 3.2. Protoplast Isolation

The isolation and genetic transformation of chestnut protoplasts were performed following the protocol described by Osakabe et al. [9], modified to suit chestnut. All solutions used for protoplast isolation are available in Supplementary Materials, File S1.

Embryogenic calli (0.1 g) obtained as described before, were used as starting material for protoplast isolation. Calli were dissected into small clumps and immediately immersed in cell-wall digestion enzyme solution containing 0.5% (*w*/*v*) macerozyme R-10 and 1% (*w*/*v*) cellulase R-10 in 20 mM morpholinoethane sulfonic acid (MES), 0.5 M mannitol, 20 mM KCl and 10 mM CaCl_2_ (pH 5.7). These enzymes allowed the cell wall degradation to release the protoplasts. To increase the digestion efficiency, explants were subjected to vacuum infiltration for 20 min and then to 4 h digestion on a rotary shaker (40 rpm at 37 °C).

After digestion, the protoplasts were filtered using a nylon mesh (100 μM) to remove cell wall debris, and an equal volume of washing solution (WS; [9]) was added to maintain the osmolarity. Protoplasts were centrifuged at 50 g for 5 min, and the supernatant was discarded. The protoplast pellet was slowly resuspended in 5 mL of WS, transferred to 5 mL of 21% (*w*/*v*) sucrose solution, and then centrifuged at 50 g for 5 min.

The ring of viable protoplasts was detected in the interface layer, then aspirated using a Pasteur pipette and resuspended in 2 mL WS. Protoplasts were again centrifuged at 50 g for 5 min, and the pellet was resuspended in 1 mL of WS solution and incubated at 4 °C for 30 min. Protoplasts were centrifuged at 50 g for 5 min, and the pellet was resuspended in 300 µL of MMG solution (solution described in Osakabe et al. [9]).

The yield of protoplasts was determined using a hemocytometer, and their viability was tested using 4% (*w*/*v*) trypan blue staining (% protoplast viability = number of observed protoplasts not stained blue / number of total protoplasts observed × 100%) [51]. Protoplasts were diluted in MMG solution to obtain a final concentration of 2 × 10^5^ in 100 µL and stored at 4 °C overnight before GFP and RNP transformation (Figure 4). Three biological replicates and three technical replicates were used to optimize the protocol.

### 3.3. Protoplast Transfection with GFP Vector

**Figure 5:**
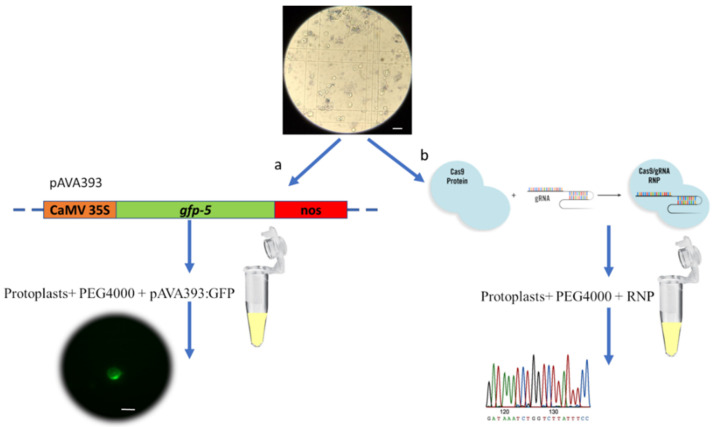
PEG-mediated transfection protocol. (**a**) Protoplast transfection using GFP marker gene and subsequent visualization using the Nikon Eclipse Ti2 fluorescent microscope. (**b**) Protoplast transfection using RNP complex by targeting the *pds* gene, followed by DNA extraction and Sanger sequencing. Scale bar = 100 µm.

The plasmid pAVA393 [52] carrying the gene coding for GFP, under the control of the 35SCaMV promoter and the Nos terminator, was used for the evaluation of protoplast transfection capacity (Figure 5). One hundred microliters of 2 × 10^5^ protoplasts were slowly mixed with 10 μg of pAVA393:GFP plasmid, followed by the addition of 100 μL of 40% (*w*/*v*) PEG The solution was slowly mixed and incubated for 10 min at room temperature. Two WS rinses were performed, and the pellet was resuspended in 1 mL of WS solution. The transfected protoplasts were incubated at 24 °C in dark conditions for 72 h before microscopy observations. After this time, the GFP signal was evaluated using a fluorescence microscope (Nikon Eclipse Ti2, Japan). The excitation was produced by an LED fluorescent source (λ = 470 nm), and the GFP emission was collected at 516 nm (Figure 5a). The transfection efficiency (%) was assessed by counting GFP fluorescing cells/number of total protoplasts observed × 100%.

### 3.4. Protoplast Transfection with CRISPR RNPs

The gene selected for setting up the transformation protocol using RNP was the *pds* gene*,* previously targeted in the first example of CRISPR/Cas9 transformation protocol in *Castanea sativa* [14]. The *pds* crRNAs(2 nmol), transactivating CRISPR RNA (tracrRNA, 2 nmol) and the Alt-R SpCas9 nuclease 3NLS (61 µM) were developed by Integrated DNA Technologies, Inc. (IDT, Coralville, IA, USA). The crRNA template sequence matched with the gRNA1 sequence (GAGTCAAGAGATGTGCTAGG) used by Pavese et al. [14].

The crRNAs and tracrRNA stocks were diluted to a final concentration of 100 µM. Appropriate concentrations of crRNA and tracrRNA were mixed with duplex buffer and annealed at 95 °C for 5 min, forming the gRNA duplex. Then, Alt-R SpCas9 3NLS nuclease and 10× PBS buffer (pH 7.4) were added. The optimized molar ratio between Cas9 and gRNA was 1:1.25.

The solution was incubated for 30 min at room temperature and then used for the transfection process. Both untransformed protoplasts and protoplasts transformed with only gRNA without the addition of the Cas9 nuclease were used as negative controls. Three biological replicates were performed to guarantee the repetitiveness of the transformation process.

One hundred microliters of the protoplast suspension (2 × 10^5^ protoplasts) were mixed with the RNP complexes, previously assembled, followed by adding 100 μL of 40% (*w*/*v*) PEG and incubation for 10 min at room temperature. Two WS rinses were performed, and the pellet was resuspended in 1 mL of WS solution and then maintained overnight in dark conditions. After overnight incubation, the solutions appeared divided into two layers. The lower layer was picked up, and the protoplasts were counted using a hemocytometer and then diluted to a final concentration of 1 × 10^6^ protoplasts/mL.

The DNA was extracted from the lower layer using EZNA^®^ Plant DNA kit (Omega Bio-tek, Norcross, GA, USA). Mutation frequencies at the *pds* target sites were evaluated through PCR amplification using primers designed on gRNA flanking regions (Table 2). DNA was amplified using KAPA HIFI Taq (KapaBiosystems, Roche, Basel, Switzerland), and the following PCR program was applied: 95 °C/3 min, followed by 30 cycles of 98 °C/20 s, 60 °C/20 s, 72 °C/45 s and 72 °C/3 min. The PCR products were purified using DNA/RNA Clean Up E.Z.N.A.^®^ kit (Omega Bio-tek, Norcross, GA, USA). Samples were sequenced using the Sanger method, and the chromatograms obtained were analyzed using the TIDE online software (https://tide.deskgen.com, accessed on 7 January 2022) (Figure 5b).

### 3.5. Protoplast Culture and Regeneration

Three induction media (named C1, C2, C3) were tested to obtain de novo organogenesis (Table 3). The protoplasts were placed in culture using the disc-culture method, which consists of protoplast inclusion in semi-solid media surrounded by the same agar-free media. Protoplast cultures were incubated in dark conditions at 24 °C. Protoplast growth was monitored weekly using the Leica-Wild Heerbrugg M8 stereoscope (Leica, Germany).

## 4. Conclusions

In conclusion, in this paper we reported an efficient protoplast isolation and transfection protocol in chestnut. Starting from embryogenic masses derived from somatic embryos as source material, the digestion in the enzyme solution consisting of 1% cellulase R10 and 0.5% macerozyme R10 allowed a high yield of isolated protoplasts (4,500,000 protoplasts/mL) with an intact spherical shape. The PEG-mediated transfection system using GFP highlights protoplast transformability. In addition, the CRISPR/Cas9 construct via RNPs was successfully applied for the first time in *Castanea sativa*, and the first transgene-free protoplasts were obtained and submitted to regeneration. Since edited protoplasts came from embryogenic calli derived from seeds, the edited genome, by this workflow, was not the one of the known and appreciated cultivar. However, the procedure defined here may constitute the basis for protoplast isolation from embryogenic calli derived from adult chestnut tree explants, and, therefore, of known genetic values. Future work on regeneration of genome-edited protoplasts will provide an opportunity to develop DNA-free genome-edited chestnut plants.

## Figures and Tables

**Table 1:** Genotyping of targeted gene mutations induced by CRISPR/Cas9 RNPs in the three transformed protoplast samples: editing efficiency, goodness-of-fit measure (R^2^), and mutations are indicated.

Samples.	Efficiency (%)	R^2^	Mutations
P1	21.4	0.96	−3; −1; +1
P2	17.9	0.95	−1
P3	14.6	0.97	−1; +1

**Table 2:** Primers used for *pds* Sanger sequencing.

Primers	
Name	Sequence
Seq_pds_gRNA1_F	TGGAAACTTTGGGTATGCATCC
Seq_pds_gRNA1_R	TTCTGTGATTGGTAGGCTTTCA

**Table 3:** Culture media tested to obtain protoplast regeneration.

Media Components	C1	C2	C3
Basal medium	MS	NN	MS
NAA (mg/L)	1	1	-
BAP (mg/L)	0.5	0.5	0.2
2,4-D (mg/L)	-	-	2
Casein hydrolysate (g/L)	0.5	-	-
D-Mannitol (g/L)	-	30	30
Sucrose (g/L)	30	5	5
L-Glucose (g/L)	50	50	50
Plant agar (g/L)	6	6	6

BAP, 6-benzyladeninepurine; 2,4-D, 2,4-dichlorophenoxyacetic acid; MS, Murashige and Skoog medium [40]; NAA, naphthaleneacetic acid; NN, Nitsch and Nitsch medium [43].

## Supplementary Materials

The following supporting information can be downloaded at: https://www.mdpi.com/article/10.3390/ijms23105762/s1.
