# Supplementary material for: First Report on Genome Editing via Ribonucleoprotein (RNP) in Castanea sativa Mill
Source: Int J Mol Sci. 2022 May 20;23(10):5762. doi: 10.3390/ijms23105762 (PMC9145500; doi:10.3390/ijms23105762)
Supplement: Supplementary file 1 [file ijms-23-05762-s001.zip › ijms-1727026-supplementary.pdf]

S1 File: solutions for protoplasts' isolation

Cell-wall digestion enzyme solution

MES: 2-[N-morpholino]-ethanesulfonic acid; $CaCl_2$: calcium chloride, KCl: potassium chloride

**Macerozyme R-10**	0,5%
**Cellulase R-10**	1%
**MES**	20 mM
**Mannitol**	0,5 M
**KCl**	20 mM
**$CaCl_2$**	10 mM

Washing solution

MES: 2-[N-morpholino]-ethanesulfonic acid; NaCl: sodium chloride; $CaCl_2$: calcium chloride, KCl: potassium chloride

**Glucose**	5 mM
**MES**	2 mM
**NaCl**	154 mM
**$CaCl_2$**	125 mM
**KCl**	5 mM

MMG solution

MgCl2: magnesium chloride

**Mannitol**	0,5 M
**MES**	4 mM
**$MgCl_2$**	15 mM
